# IL21 inhibits miR-361-5p to promote MAP3K9 and further aggravate the progression of shoulder arthritis

**DOI:** 10.18632/aging.205793

**Published:** 2024-05-07

**Authors:** Kangning Hao, Pengchao Lin, Jing Li, Jie Hu, Jiangyong Wang, Fei Li

**Affiliations:** 1 Department of Orthopedic Surgery, The Third Hospital of Shijiazhuang, Shijiazhuang 050011, Hebei, P.R. China; 2 Department of Nursing, Hebei Province Eighth People’s Hospital, Shijiazhuang 050011, Hebei, P.R. China; 3 Department of Radiology, The Third Hospital of Shijiazhuang, Shijiazhuang 050011, Hebei, P.R. China

**Keywords:** shoulder arthritis, IL21, miR-361-5p, MAP3K9, MMPs

## Abstract

Abstract: Objective: This research aimed to explore IL-21/miR-361-5p/MAP3K9 expression in shoulder arthritis and identify its regulatory pathways.

Methods: We established a rat shoulder arthritis model, then quantified IL21 and miR-361-5p in synovial fluid using ELISA and monitored the arthritis development. Additionally, IL21’s effect on miR-361-5p levels in cultured human chondrocytes (HC-a) was assessed. Chondrocyte cell cycle status and apoptosis were measured via flow cytometry. Interactions between miR-361-5p and MAP3K9 were confirmed through dual-luciferase reporting and bioinformatic scrutiny. Protein levels of MAP3K9, p-ERK1/2, p-NF-κB, MMP1, and MMP9 were analyzed by Western blots.

Results: IL21 levels were elevated, while miR-361-5p was reduced in the synovial fluid from arthritic rats compared to healthy rats. IL21 was shown to suppress miR-361-5p in chondrocytes leading to hindered cell proliferation and increased apoptosis. Western blots indicated that miR-361-5p curbed MAP3K9 expression, reducing MMP activity by attenuating the ERK1/2/NF-κB pathway in chondrocytes.

Conclusion: IL21 upregulation and miR-361-5p downregulation characterize shoulder arthritis, resulting in MAP3K9 overexpression. This chain of molecular events boosts MMP expression in chondrocytes and exacerbates the condition’s progression.

## INTRODUCTION

Osteoarthritis (OA), a progressive joint disorder, is marked by articular cartilage deterioration, bone changes, and synovial inflammation, leading to symptoms such as joint pain and compromised function. Currently, OA lacks disease-altering drug treatments and is managed through non-surgical interventions like lifestyle modifications and palliative measures until potential joint replacement. This condition mainly arises from age-related cartilage degradation, subchondral bone hardening, bone cysts, and subsequent synovitis [1, 2]. Symptoms are more prevalent in those over 60, with pain, swelling, and stiffness being primary complaints [3]. Defective chondrocyte biology within the joint is a key contributor to the condition’s onset, but the exact pathogenesis remains undetermined [4–6] and may involve chondrocyte loss due to inflammation.

IL21, mainly produced by CD4+ T cells and also by T follicular helper and Th17 cells, is a type 1 cytokine. It has a 4-helix structural domain resembling IL2, IL4, and IL15, with its 162aa precursor consisting of a 31aa signal and a 131aa mature protein [7–9]. Its elevated secretion under various inflammatory states suggests a link with arthritis development, as IL21 binds to its receptor IL21R [10]. Moreover, IL21 regulates miRNAs like miR-29 and miR-146a, implicated in disease modulation [11, 12].

MiRNAs, short 18–24 nucleotide RNAs, act as post-transcriptional regulators of gene expression impacting multiple biological pathways and disease states. MiR-361-5p, in particular, has been identified as critical in chondrocyte functionality and joint degeneration [13]. MAP3K9, an initiator of the MAPK/JNK signaling cascade, is instrumental in apoptosis regulation. The MAPK family includes the ERK1/2, JNK, and p38 MAPK subgroups [14]. Previous studies have identified MAP3K9 as miR-148b’s target and found its interaction site with miR-1247 [15, 16]. In our research, we utilized bioinformatics to investigate potential miR-361-5p binding sites on MAP3K9, assessed IL21’s impact on miR-361-5p, and explored how miR-361-5p interacts with MAP3K9 to affect chondrocyte function in shoulder arthritis.

## METHODS

### Prediction of MiRNA binding to genes

The miRNAs binding of key genes was studied using the Targetscan database (http://www.targetscan.org/). The miRNA-mRNA was predicted by the miRNA-targeting base gene sub-method, and the key miRNAs of the target genes were predicted by this method.

### Animals

Male Wistar rats aged 10 weeks and weighing 220–250 g were used. All rats were housed under standard laboratory conditions (temperature 24°C , 12 h light/dark cycle) and given standard food and water. This study was conducted in accordance with all the guidelines of the Third Hospital of Shijiazhuang and was approved by the Medical Ethics Committee of the Third Hospital of Shijiazhuang. The Ethics lot number is 2018-006.

### Construction of rat shoulder arthritis model

Animals were randomly grouped (*n* = 6 in each group). Each rat underwent anesthesia via intraperitoneal injection using sodium pentobarbital, ensuring aseptic conditions. A singular intra-articular injection of 0.3 mg of monoiodoacetic acid (MIA) was administered to the left shoulder (sourced from Sigma-Aldrich, St. Louis, MO, USA). Additionally, for comparison, the left shoulder joints of control rats not receiving MIA treatment were assessed to identify any potential changes prompted by the temporary elevation of intra-articular pressure due to the saline injection. The rats were then randomly divided into four groups: the normal group, the shoulder arthritis group, the miR-NC group, and the miR-361-5p mimics group.

### Cell culture and transfection

Human chondrocytes HC-a (2 × 10^5^; American Type Culture Collection, Manassas, VA, USA) were cultivated in DMEM medium enriched with 10% FBS, penicillin (100 IU/ml), and streptomycin (100 IU/ml) within an environment maintained at 37°C, with 5% CO_2_ and a humidity of 70%. Upon attaining 90% confluence, these cells were subcultured, and those in logarithmic growth phase were selected for use in experiments. HC-a cells were grouped as follows: a normal control group, an IL21-stimulated group, the IL21+miR-NC group, and the IL21+miR-361-5p group. MiR-NC (5′-UUC UCC GAA CGU GUC ACG UTT-3′), miR-361-5p (5′-ACG CCU GGA GAU UCA UAU AAU AAU AU-3′).

For the transfection process, HC-a cells in their logarithmic growth phase (2 × 10^5^) were seeded in a 24-well plate with 10% FBS in DMEM medium devoid of antibiotics. Once 70% confluence was reached, transfection commenced. A miR-361-5p mimic solution (20 pmol/μl; Sangon Biotech, Shanghai, China) was prepared by combining 1.25 μl of the mimic with 50 μl of Opti-MEM I medium (Thermo Fisher Scientific, Waltham, MA, USA). Concurrently, 1 μl of Lipofectamine 3000 (Thermo Fisher Scientific) was mixed with 50 μl of Opti-MEM. The contents of both vials were combined after a 5-minute incubation and then allowed to incubate for an additional 20 minutes at room temperature before adding to the cells. Plasmids for MAP3K9, miR-NC + MAP3K9, and miR-361-5p mimic + MAP3K9 were transfected into appropriate cells using Polyplus transfection reagent (Invitrogen; Thermo Fisher Scientific), in accordance with the manufacturer’s guidelines. Experiments proceeded 3 hours post transfection. Moreover, in the experimental groups, cells were treated with an ERK1/2 agonist Yoda1 (3 μM) and an NF-κB agonist Diprovocim (5 nM), with subsequent procedures occurring after.

### Hematoxylin-eosin (HE) staining

Tissue preparation involved fixing in 10% formaldehyde for two days, dehydration through a series of ethanol washes, clearing in xylene, embedding in paraffin, and sectioning into slides of 5 μm thickness. Slides were stained using hematoxylin and eosin (HE) and then sealed with neutral balsam. Observations were made under a 200× magnification fluorescence microscope.

### Quantitative real-time polymerase chain reaction (qRT-PCR)

RNA extraction was conducted using TRIzol reagent. Peripheral serum (150 μl) and cells (1 × 10^6^) were treated with 750 μl and 1ml of TRIzol LS respectively at room temperature following the provided protocol (Walton Technology, USA). From 1μg of RNA, cDNA synthesis occurred and was preserved at −20°C. The reverse transcription mix included RNA template, 2× Buffer Mix, 0.1% BSA, miScript II RT enzyme Mix, and water. The reaction proceeded at 37°C for one hour, followed by dilution with RNase-free water up to 50 μl. qRT-PCR included a mixture of cDNA, primers, and ddH2O. The thermal cycle involved an initial denaturation, followed by 40 cycles of denaturation and annealing (95°C for 30 s, and 60°C for 30 s, respectively). The primer sequences of miR-361-5p are 5′-TTA TCA GAA TCT CCA GGG GTA C-3′ (forward) and 5′-AAA TTG TAT AAA GAG AAA TT-3′ (reverse). The primer sequences for U6 were 5′-CTC GCT TCG GCA GCA CA-3′ (forward) and 5′-AAC GCT TCA CGA ATT TGC GT-3′ (reverse). The 2^−ΔΔCt^ method was used to calculate the relative expression of miR-361-5p against U6. Each sample was tested in triplicate.

### Enzyme-linked immunosorbent assay (ELISA)

Standard IL21 and synovial fluid from rat shoulder joints (100 μl/well) were added to an ELISA plate pre-coated with specific antibodies, followed by incubation with the conjugate at 37°C for one hour. The plate underwent five washing steps, and then substrates A and B were applied (50 μl/well each). The reaction was halted by adding stop solution (50 μl/well), and absorbance readings were taken subsequently.

### CCK-8 detection

In a separate assay, chondrocytes were seeded in a 96-well plate at a density of 2000 cells per well. Cell viability was assessed at 24, 48, and 72 hours by introducing 20 μl of CCK-8 reagent (Beyotime, Shanghai, China) to each well, followed by incubation with CCK-8 solution (150 μl) at 37°C for two hours. The optical density of each well was recorded at 490 nm to construct the growth curves. Triplicate wells were sampled for each condition to compute an average value.

### Flow cytometry

For the cell cycle analysis, cells treated with miR-361-5p mimics and miR-NC for a day were cleaned with cold phosphate-buffered saline twice. The BD Cell Assay^™^ Plus DNA Kit was employed as per the instructions provided. The protocol involved treating cells with solutions A, B, and C sequentially, each for ten minutes, with the last step conducted in darkness before analyzing with flow cytometry, and interpreting results with ModFit software version 3.2. Furthermore, cells, post-IL21 exposure for 24 hours, were prepared identically and analyzed for apoptosis rates using the ANXN V FITC Apoptosis DTEC Package I in accordance with the manufacturer’s protocol. Populations were identified as early apoptotic (Annexin V-positive), necrotic (propidium iodide-positive), and late apoptotic (double-positive) by flow cytometry analysis.

### Western blotting experiments

At 48 hours after transfection, HC-a cells went through a double wash with phosphate-buffered saline and were lysed on ice for five minutes with 600 μl of RIPA buffer supplied by the Shanghai Institute of Biotechnology, China. The lysates were then centrifuged at 12,000 rpm at 4°C for ten minutes, and the supernatant’s protein content was quantified using the Bicinchoninic acid (BCA) assay kit from TransGen Biotechnology Co., Ltd., Beijing, China. Following this, protein samples were mixed with loading buffer, heated for ten minutes for denaturation, and loaded on a 10% SDS-PAGE gel for electrophoresis at 100 V. Proteins were subsequently transferred to PVDF membranes, blocked with non-fat milk. These membranes were then incubated with primary antibodies to MAP3K9 (1:1000, Abcam, ab228752), phosphorylated ERK1/2 (1:1000, Abcam, ab201015), phosphorylated NF-κB (p-NF-κB, 1:1000, Abcam, ab76302), as well as MMP1 (1:1000, Abcam, ab137332), MMP9 (1:1000, Abcam, ab283575), and GAPDH (1:10,000, Abcam, ab9484) overnight at 4°C. After three thorough 15-minute washes with Tween 20 enhanced PBS, the blots were incubated with a goat anti-rabbit horseradish peroxidase-linked secondary antibody (1:4000, Cambridge, UK) for an hour at room temperature and then washed again thrice. Detection of the proteins was done using an enhanced chemiluminescence kit (Abcam, Cambridge, UK) and imaging signals were captured and quantified with Image Lab software version 3.0 (Bio-Rad, Hercules, CA, USA).

### Statistical analysis

In statistical processing, data were expressed as mean ± standard deviation and analyzed using Prism 9.0 software. A paired Student’s *t*-test was applied for comparisons between two groups, while one-way ANOVA followed by Student-Newman-Keuls post hoc tests were utilized for multi-group analyses. A *p*-value less than 0.05 indicated statistical significance.

## RESULTS

### Bioinformatics analysis results

**Figure 1:**
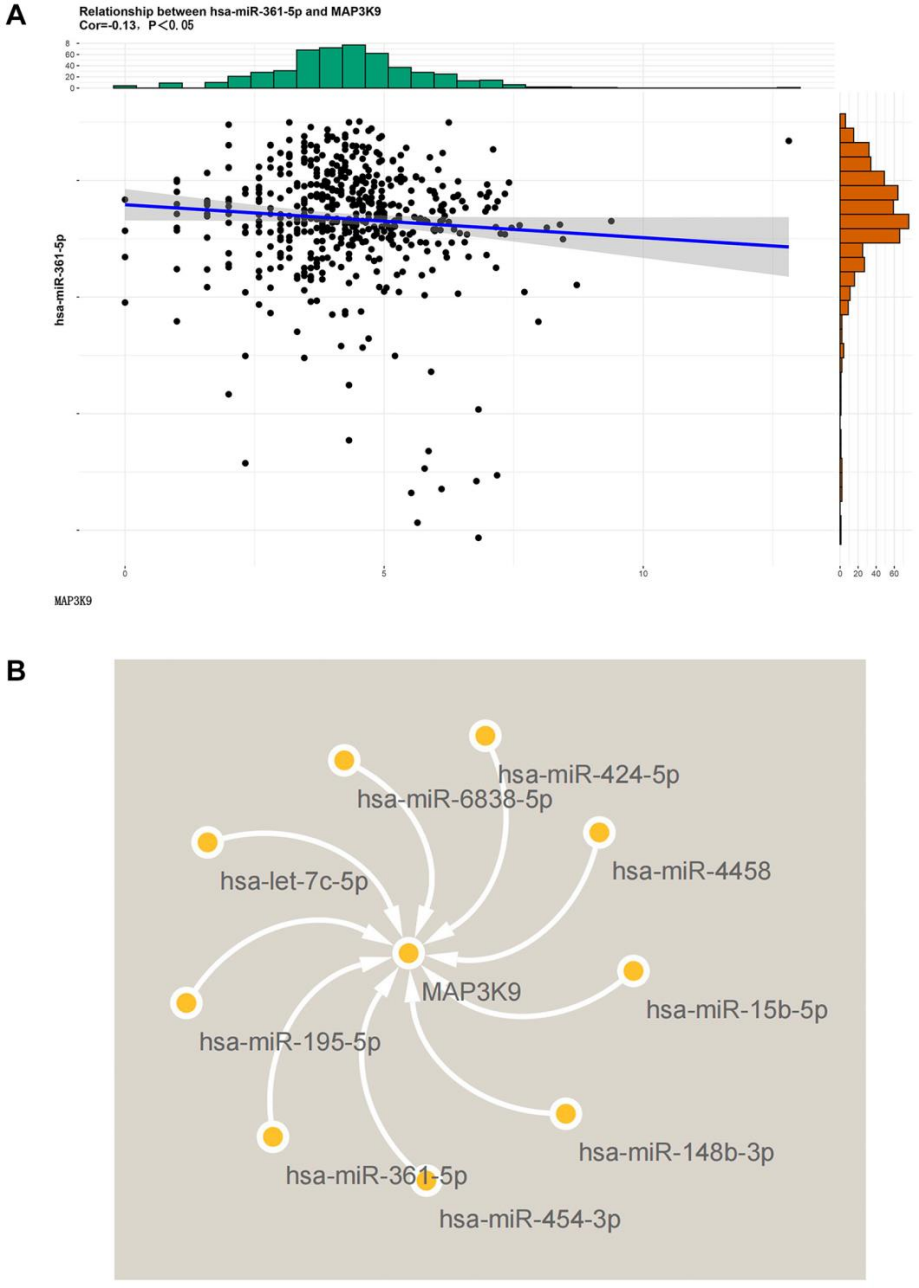
**Diagram of the results of bioinformatics analysis.** (**A**) The correlation between MAP3K9 and miR-361-5p was analyzed based on the Targetscan database. MAP3K9 was negatively correlated with miR-361-5p. (**B**) Graph of MAP3K9-related miRNAs based on Cytoscape software.

The key miRNAs of MAP3K9 were predicted based on the Targetscan database, and the correlation between the two was analyzed based on the correlation (Figure 1A), and the regulatory map between MAP3K9 and upstream miRNAs was drawn based on Cytoscape software (Figure 1B), and the results showed that MAP3K9 was significantly negatively correlated with multiple miRNAs including hsa-miR-361-5p. These results suggest that MAP3K9 is negatively regulated by hsa-miR-361-5p.

### IL21 exerting a negative regulatory effect on miR-361-5p in chondrocytes

**Figure 2:**
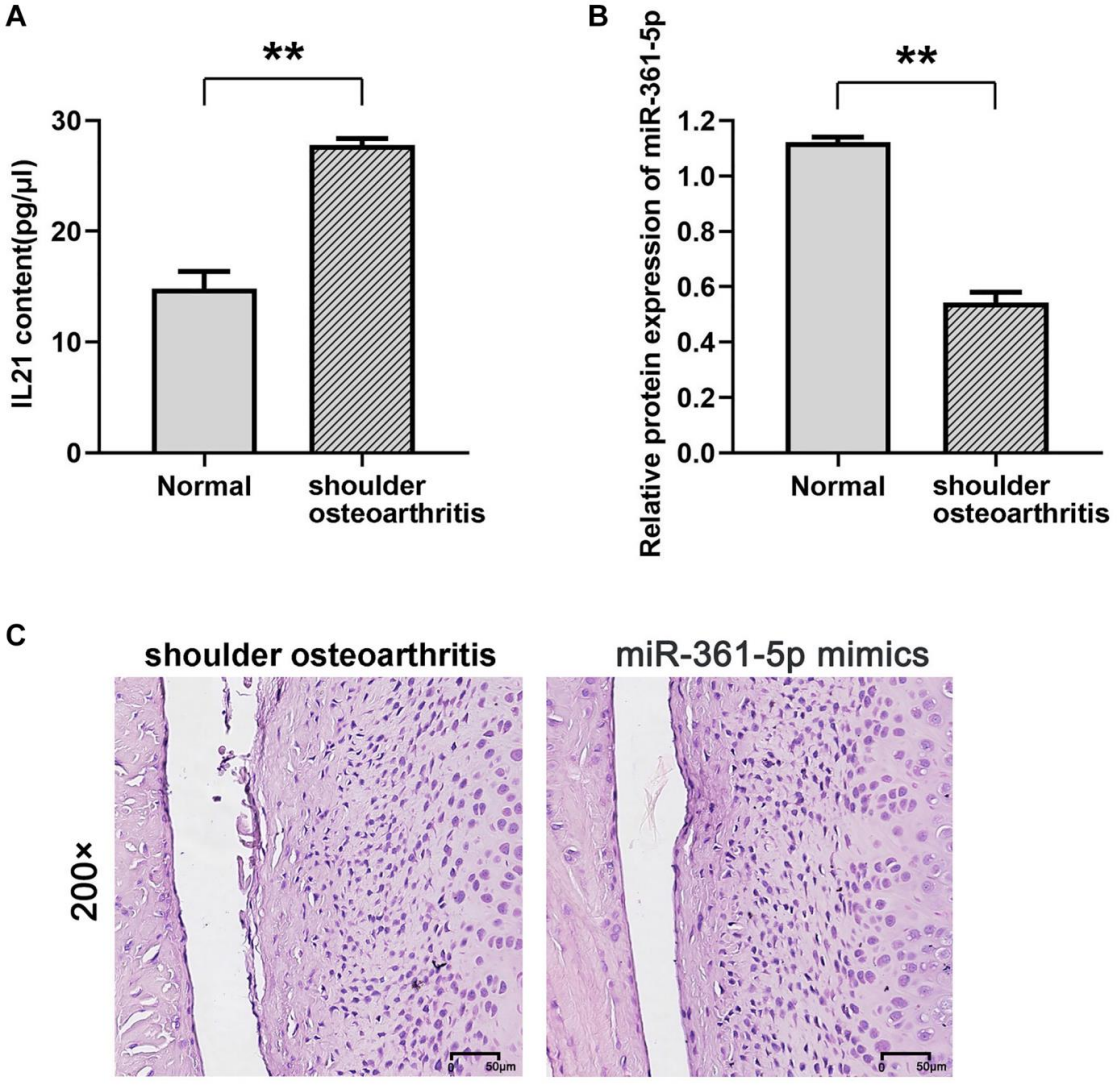
**Expression of IL21 and miR-361-5p and their possible relationship.** (**A**, **B**) ELISA was used to detect the expression of IL21 in the shoulder joint effusion of rats with normal shoulder and rats with arthritis. (**B**) qRT-PCR was used to detect the expression of miR-361-5p in shoulder joint effusion in rats with normal shoulder and rats with arthritis. (**C**) HE staining was used to detect the effect of miR-361-5p on the pathological condition of shoulder joint cavity in arthritic rats. *N* = 8; ^**^P < 0.01, Normal vs. shoulder arthritis.

Quantitative evaluations via ELISA for IL21 and qRT-PCR for miR-361-5p confirmed these findings (Figure 2A, 2B). The miR-361-5p mimic group showed a decrease in inflammation and less cartilage erosion compared to the group with shoulder arthritis, as demonstrated by histological staining (Figure 2C). In this model, miR-361-5p appeared to play a therapeutic role by mitigating the disease progression.

### IL21 and miR-361-5p regulate chondrocyte proliferation

**Figure 3:**
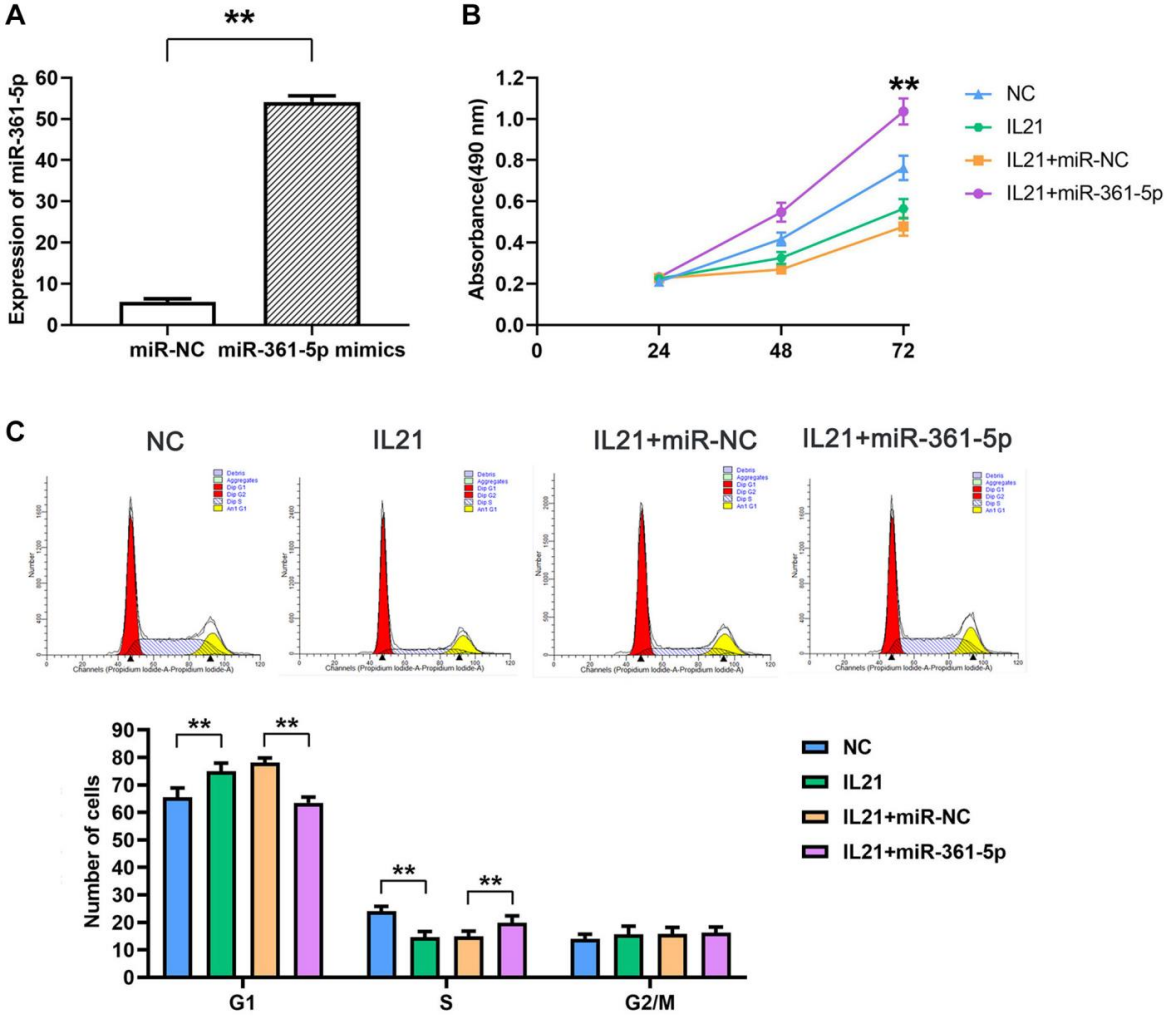
**IL21 inhibits chondrocyte proliferation by regulating the expression of miR-361-5p.** (**A**)The expression of miR-361-5p in HC-a cells in the NC group and miR-361-5p mimics group was verified to verify the successful transfection of miR-361-5p mimics. (**B**) Proliferative capacity of HC-a cells after co-treatment with IL21 or IL21 and miR-361-5p.miR-361-5p can promote the proliferative ability of HC-a cells. (**C**) Cell cycle analysis of HC-1 cells after co-treatment with IL21 or IL21 and miR-361-5p. miR-361-5p can promote the S phase of HC-a cells stimulated by IL21. *N* = 8; ^**^P < 0.01: miR-NC vs. miR-361-5p mimics; NC vs. IL21; IL21+miR-NC vs. IL21+miR-361-5p.

Experimental modifications in HC-a cell lines showed augmented expression of miR-361-5p post-transfection with mimics, in contrast to the control group (*P* < 0.05) (Figure 3A). Cell viability assays indicated that IL21 attenuated cellular proliferation after 48 and 72 hours, whereas cells transfected with miR-361-5p mimics exhibited increased proliferation when co-treated with IL21, compared to cells treated with IL21 and a miRNA negative control (*P* < 0.05) (Figure 3B). Further cell cycle analysis by flow cytometry suggested that IL21 influenced cell cycle progression by increasing the proportion of G1-phase cells and decreasing S-phase cells, a trend reversed by miR-361-5p co-treatment (*P* < 0.05) (Figure 3C).

### miR-361-5p alleviated IL21-promoted chondrocyte apoptosis

**Figure 4:**
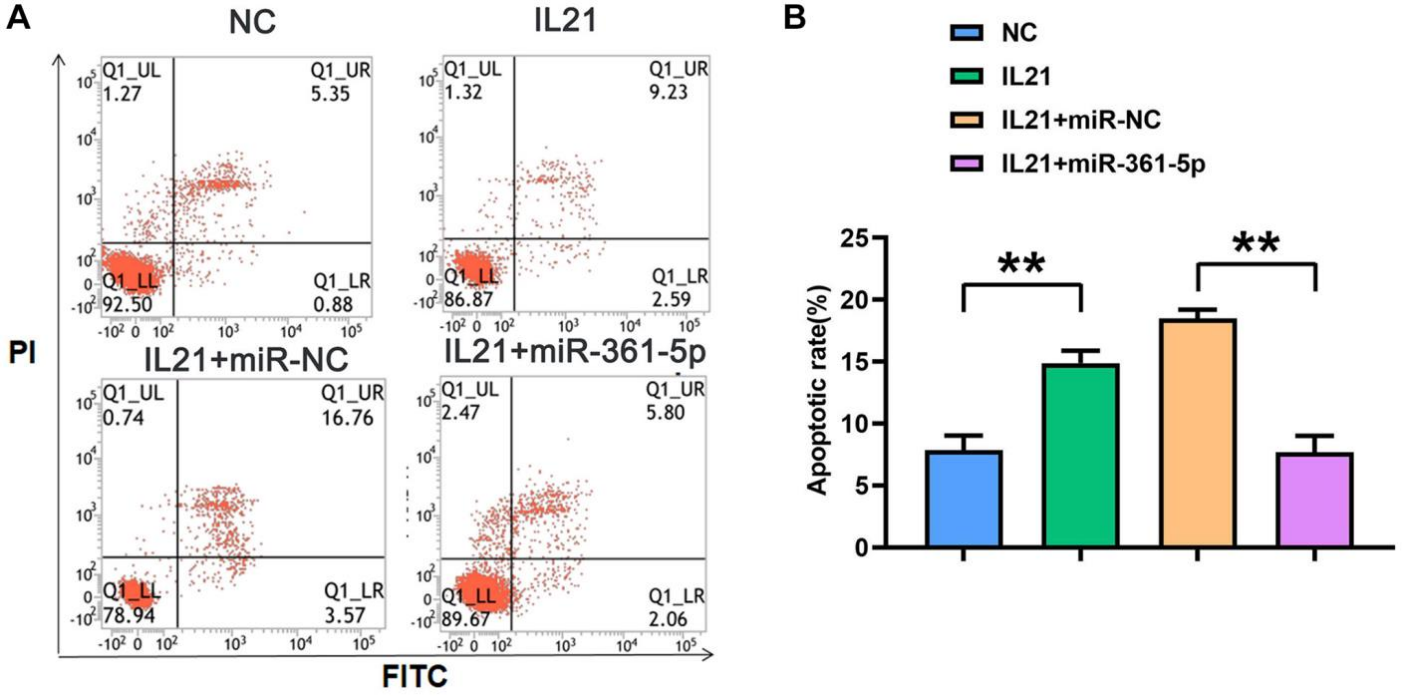
**IL21 promotes chondrocyte apoptosis by regulating the expression of miR-361-5p.** (**A**) Plots of apoptosis results of HC-1 cells after co-treatment with IL21 or IL21 and miR-361-5p. (**B**) The apoptosis rate of HC-1 cells after co-treatment with IL21 or IL21 and miR-361-5p. *N* = 8; ^**^P < 0.01, NC vs. IL21, IL21+miR-NC vs. IL21+miR-361-5p.

An additional facet of regulation by IL21 and miR-361-5p was observed in apoptosis studies. HC-a cells under IL21 stimulation presented an increased apoptosis rate; however, cells co-treated with miR-361-5p showed a lower rate of apoptosis under the same conditions (*P* < 0.05) (Figure 4), indicating the protective effect of miR-361-5p against IL21-induced chondrocyte apoptosis.

### MiR-361-5p is associated with MAP3K9

**Figure 5:**
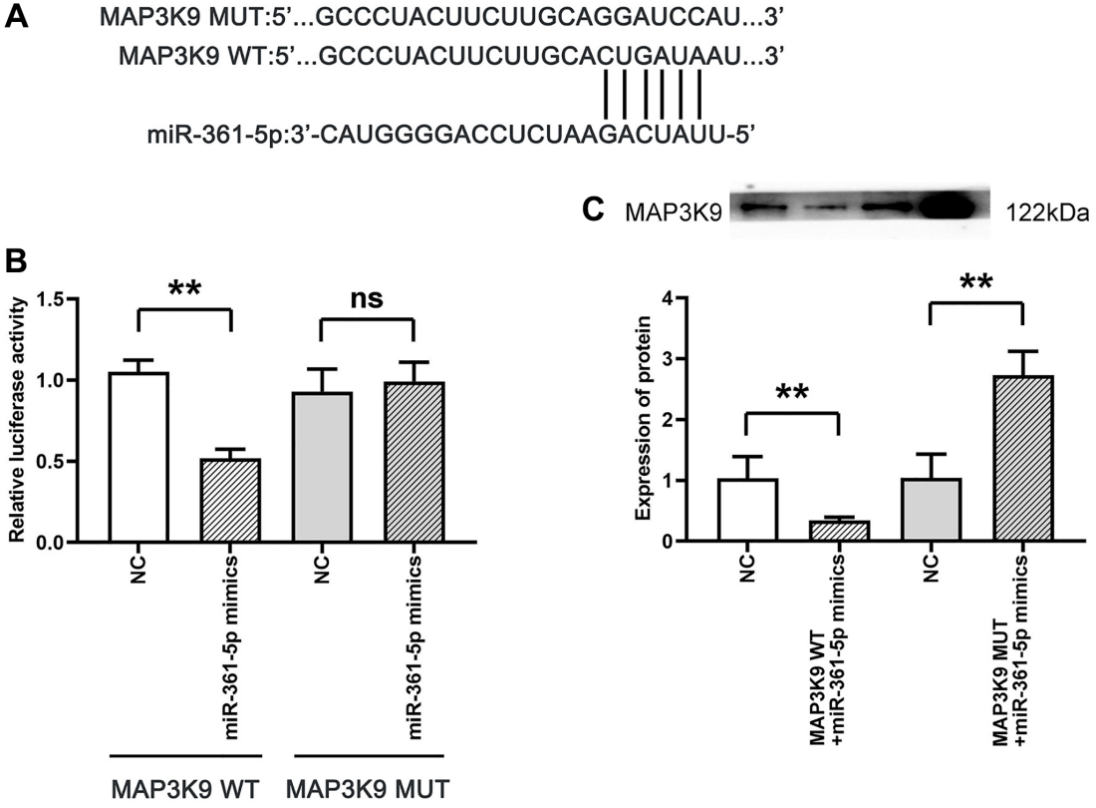
**Association between MAP3K9 and miR-361-5p.** (**A**) Prediction of MAP3K9 targeting by miR-361-5p using TargetScan. (**B**) The association between miR-361-5p and MAP3K9 was verified by the dual luciferase reporter gene assay. (**C**) Protein band diagram and relative protein expression statistics of MAP3K9. *N* = 3; ^**^P < 0.01, MAP3K9 WT+NC vs. MAP3K9 WT+miR-361-5p mimics, NC vs. MAP3K9 WT+miR-361-5p mimics, NC vs. MAP3K9 MUT+miR-361-5p mimics; ^ns^*P* > 0.05, MAP3K9 MUT+NC vs. MAP3K9 MUT+miR-361-5p mimics.

Our research demonstrated an interaction between miR-361-5p and MAP3K9, with a specific focus on MAP3K9 for its integral role in apoptosis via the MAPK/JNK pathway and its relatively unknown function in the context of shoulder arthritis. Evidence of this interaction was shown through dual-luciferase reporter assays; We constructed reporter genes with either the wild-type (wt) or mutated (mut) 3′-UTR of MAP3K3 into the pmiR luciferase vector and cotransfected them with miR-361-5p mimics or control constructs into HC-a cells. The cotransfection with miR-361-5p mimics and the wt MAP3K9 3′-UTR reporter significantly reduced luciferase activity, while the mutant 3′-UTR did not show this reduction. Western blot analysis supported that the MAP3K9 protein levels were reduced in the miR-361-5p mimics+MAP3K9 WT group compared to the control, but this effect was not observed with the mimic+MAP3K9 MUT group (Figure 5), thus verifying MAP3K9 as a miR-361-5p target.

### Overexpression of miR-361-5p inhibits the expression of MAP3K9, thereby suppressing the expression of MMPs in chondrocytes

**Figure 6:**
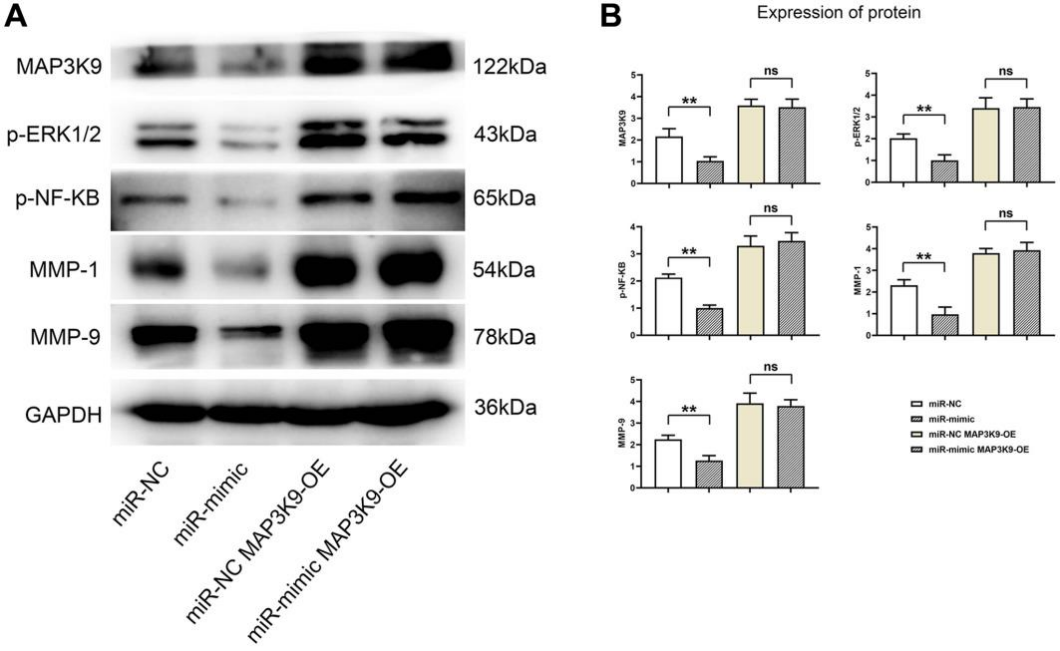
**Overexpression of miR-361-5p inhibits the expression of MAP3K9, thus inhibiting the expression of MMPs in chondrocytes.** (**A**) Protein band plots of MAP3K9, p-ERK1/2, p-NF-κB, MMP1 and MMP9. (**B**) Relative protein expression statistics of MAP3K9, p-ERK1/2, p-NF-κB, MMP1 and MMP9. *N* = 3; ^**^P < 0.01, miR-NC vs. miR-mimic; ^ns^*P* > 0.05, miR-NC+MAP3K9 OE vs. miR-mimic+MAP3K9 OE.

When testing the effects of miR-361-5p on chondrocytes, it was discovered that miR-361-5p overexpression hindered MAP3K9, which further suppressed MMP1 and MMP9 levels, regulators of extracellular matrix degradation. This suppressive effect, evidenced by lower levels of key signaling proteins and MMPs in miR-361-5p overexpressed cells, could be counteracted by MAP3K9 overexpression, suggesting that miR-361-5p’s impact is mediated through MAP3K9 (Figure 6).

### Overexpression of miR-361-5p inhibits the expression of MMPs by inhibiting the ERK1/2/NF-κB signaling pathway

**Figure 7:**
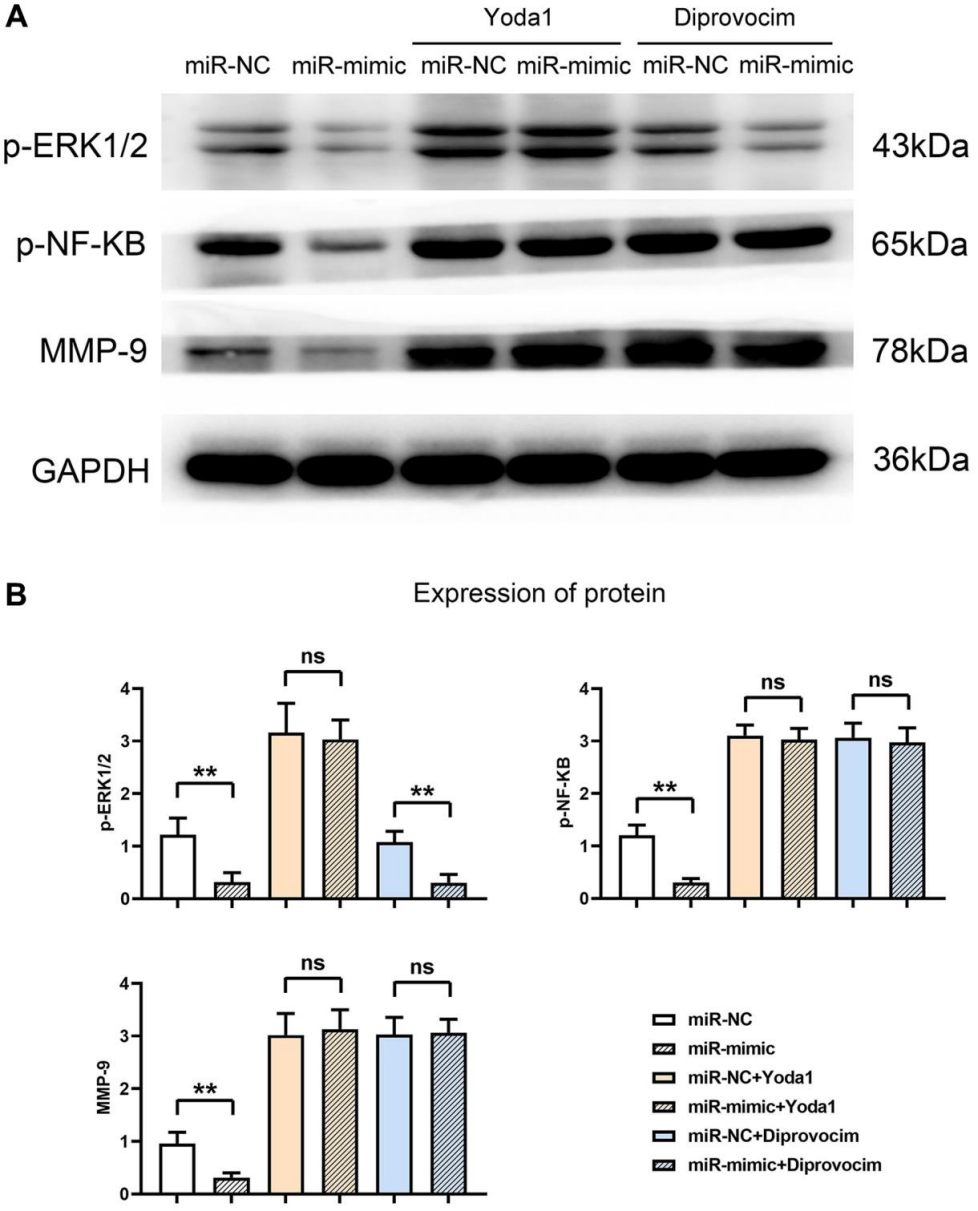
**Overexpression of miR-361-5p inhibits the expression of MMPs by inhibiting the ERK1/2/NF-κB signaling pathway.** (**A**) Protein band plots of p-ERK1/2, p-NF-κB and MMP9. (**B**) Relative protein expression statistics of p-ERK1/2, p-NF-κB and MMP9. *N* = 3; ^**^P < 0.01, miR-NC vs. miR-mimic, miR-NC+Diprovocim vs. miR-mimic+Diprovocim; ^ns^*P* > 0.05, miR-NC+Yoda1 vs. miR-mimic+Yoda1, miR-NC+Diprovocim vs. miR-mimic+Diprovocim.

**Figure 8:**
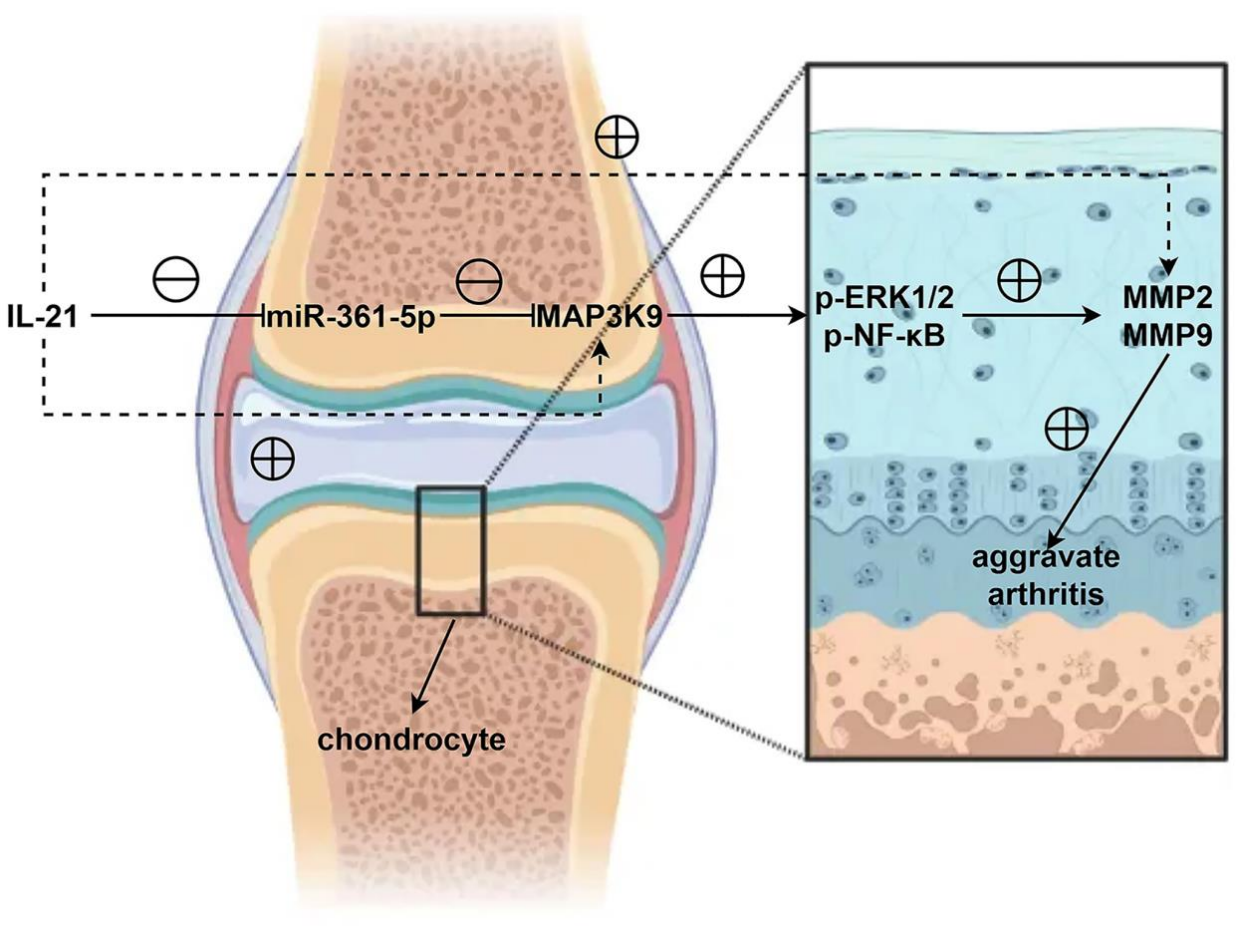
In shoulder arthritis, IL21 can inhibit the expression of miR-361-5p in chondrocytes thereby mediating MAP3K9 in chondrocytes to promote the activation of the ERK1/2/NF-κB signaling pathway, further inhibiting the expression and secretion of MMPs and aggravates the progression of shoulder arthritis.

Further exploration revealed that miR-361-5p overexpression resulted in diminished levels of activated ERK1/2 and NF-κB signaling molecules, along with MMP9. These levels were restored upon adding ERK1/2 or NF-κB agonists, aligning the protein levels of the miR-mimic group with the control group, as shown in our experimental outputs (Figure 7). The detailed data supporting these conclusions, including *P*-values and associated statistical information, can be found in Supplemental Data 1. This series of experiments supports the role of miR-361-5p in attenuating the ERK1/2/NF-κB pathway through MAP3K9 modulation and subsequently reducing MMP production (Figure 8).

## DISCUSSION

Osteoarthritis (OA) is a widespread chronic joint condition commonly seen in the elderly, particularly in individuals aged 65 and above [17, 18]. OA predominantly affects the knee, hand, hip, and spinal joints, leading to significant mobility impairments as a primary musculoskeletal cause in the aged population [19]. Various risk factors such as genetics, aging, obesity, and misaligned joints have been linked to OA, but its pathogenesis is not fully understood [4, 5]. The markers of OA include persistent pain, joint instability, stiffness, deformity, and reduced joint space depicted on radiographic images.

Normal wear or disease-related processes can damage articular cartilage. Initially, OA may manifest with a superficially intact cartilage layer, but subsequent changes in the extracellular matrix’s molecular makeup occur [20]. Due to their limited ability to regenerate and typically low metabolic activity, articular chondrocytes respond to damage by briefly proliferating and increasing matrix synthesis in an effort to repair, which involves chondrocyte clustering and a shift towards hypertrophy, denoted by markers such as Runx2, ColX, and Mmp13. As articular cartilage degrades with the breakdown of its proteoglycan and collagen framework [21], joint cartilage integrity deteriorates, eventually leading to cartilage loss and bone friction in the joint, causing pain and restricted movement. Complications like subchondral bone sclerosis, cysts, osteophyte formation, and muscle and tendon weakening can also arise.

IL21, a pro-inflammatory cytokine principally produced by immune cells, plays a pivotal role in inflammatory and autoimmune conditions like arthritis and systemic lupus erythematosus (SLE) [22, 23]. The function of IL15 in rheumatoid arthritis, which correlates with autoantibody activity, is also well-documented [24]. However, IL21’s role in osteoporosis linked to shoulder joint degeneration remains unreported. This study notes elevated levels of IL21 in the synovial fluid of shoulder arthritis in rats, indicating a role in disease exacerbation.

In addition to cytokines, miRNA molecules play a role in bone and joint inflammation. MiR-155 is implicated in rheumatoid arthritis by influencing immune cells [25], while miR-133a and miR-365a-3p are associated with osteoporosis by affecting osteoclast differentiation and inhibiting osteogenic differentiation, respectively [26–28]. This research found reduced miR-361-5p in the synovial fluid of arthritic shoulder joints in rats, with an inverse relationship to IL21, suggesting IL21 may exert effects through miR-361-5p.

The study further examined the impact of IL21/miR-361-5p on chondrocytes, which are vital to maintaining joint health [29]. Chondrocyte malfunction, such as atypical collagen production, proliferation, and apoptosis, is a prime contributor to arthritis [30, 31]. IL21 was found to suppress HC-a chondrocyte proliferation, promote apoptosis, and downregulate miR-361-5p. The restoration of HC-a cell functions was noted with increased miR-361-5p, implicating MAP3K9 as a target gene, and revealing a role for miR-361-5p in regulating MMP1, a collagen-degrading enzyme [32]. The ERK1/2/NF-κB signaling pathway’s involvement in MMP expression was confirmed using specific agonists.

In summary, this study highlights the significance of the IL21/miR-361-5p/MAP3K9 axis in shoulder arthritis pathology. IL21 downregulates miR-361-5p in chondrocytes, facilitating MAP3K9’s action on the ERK1/2/NF-κB pathway, which in turn suppresses MMP secretion, exacerbating shoulder arthritis.
